# Valorization of corn silk protein: pH-driven structural modulation for emulsion-templated oleogel fabrication

**DOI:** 10.1016/j.crfs.2026.101607

**Published:** 2026-10-22

**Authors:** Chuxin Ke, Jiayang He, Fanyu Hao, Bo Pang

**Affiliations:** Department of Food Science and Technology, Faculty of Science, National University of Singapore, 117543, Singapore

**Keywords:** Plant protein, Corn silk, pH, Structure, Emulsion-templated, Oleogel

## Abstract

Corn silk, an underutilized agricultural by-product, represents a promising yet largely unexplored source of plant proteins. In this study, corn silk protein was extracted via alkaline solubilization-acid precipitation, and its structural and functional properties were systematically investigated through pH modification (pH 3-11). Scanning electron microscopy revealed that control corn silk protein consisted of relatively uniform sub-spherical particles with diameters of 1.5 to 1.6 μm, while secondary structure analysis indicated that β-sheet and β-turn were the dominant conformations. Furthermore, pH 11 treatment was associated with improved solubility and emulsifying performance, which may contribute to enhanced interfacial stabilization. Specifically, C11 showed higher solubility (75.8%), emulsifying activity index (15.11 m^2^/g), and emulsifying stability index (214.11 min) than the control corn silk protein and other groups, resulting in improved emulsion stability. Corn silk protein-based oleogels with various water-to-oil volume ratios (5/5, 4/6, 3/7, 2/8, 1/9) were prepared using an emulsion-templated method (oven-drying at 40 °C for 24 h). At a water-to-oil volume ratio of 3/7, corn silk protein formed a relatively uniform protein network that effectively entrapped and stabilized the oil phase, resulting in an oleogel with a soft-solid structure, enhanced hardness, and superior oil-holding capacity (98%). This study suggests the feasibility of extracting proteins from corn silk and subsequently using them to prepare corn silk protein-based oleogels.

## Highlights

• Corn silk protein was valorized as a plant protein for oleogel fabrication.
• Alkaline treatment at pH 11 enhanced protein solubility and emulsifying properties.
• A 3:7 water-to-oil ratio resulted in a more uniform oleogel network.
• Links among protein structure, interface, and oleogel formation were proposed.

## Graphical abstract

**Figure:**
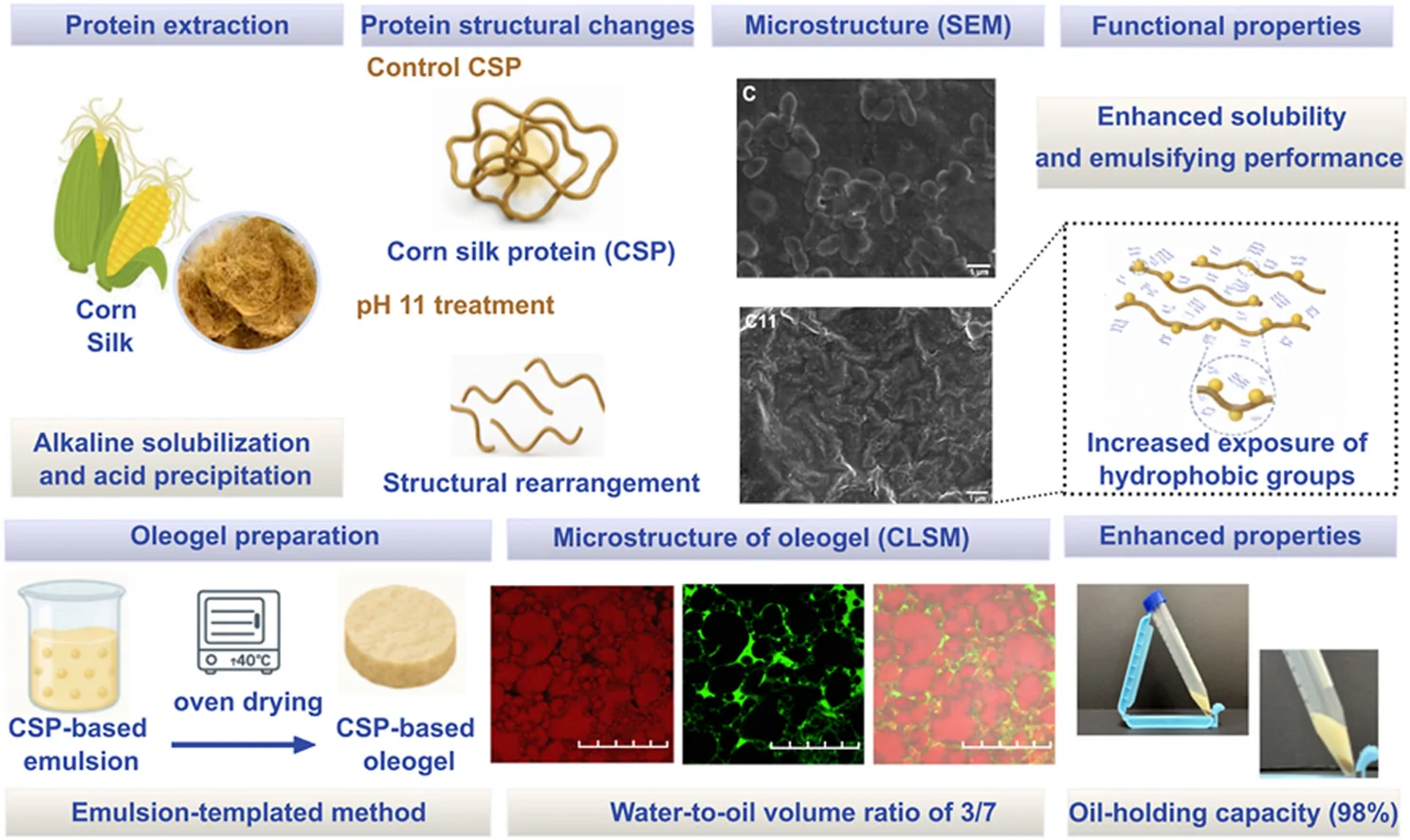

## 1 Introduction

The increasing global demand for healthier lipid profiles in food products has driven considerable interest in strategies to replace saturated and *trans* fats with structured liquid oils that retain desirable textural and sensory attributes. Oleogels, semi-solid systems in which liquid oils are immobilized within three-dimensional networks formed by oleogelators, have emerged as one of the most promising solutions to this challenge (Li et al., 2025, Li et al., 2025). By converting liquid vegetable oils into viscoelastic, solid-like materials, oleogels can replicate the mechanical and organoleptic properties of conventional solid fats such as butter, margarine, and shortening, while providing a healthier lipid profile (Gundogan et al., 2026). Among the various oleogel fabrication strategies, the emulsion-templated method has attracted particular attention due to its simplicity and high controllability. In this approach, an oil-in-water emulsion is first prepared using a suitable emulsifier, then dehydrated to remove the aqueous phase, yielding the final oleogel. Compared with freeze-drying, oven drying offers distinct advantages for the dehydration step, including lower energy consumption, simpler equipment requirements, and avoidance of ice-crystal-induced structural damage, thereby facilitating the formation of compact and continuous gel networks suitable for large-scale food manufacturing (Caldas-Abril et al., 2025).

For emulsion-templated oleogels, the selection of the emulsifier is of paramount importance, as it governs both emulsion stability and the formation of the resulting gel network. Plant proteins have garnered significant attention in this context owing to their amphiphilic nature, which enables spontaneous adsorption at oil-water interfaces to form cohesive interfacial films, and their alignment with growing consumer demand for clean-label, sustainable, and allergen-conscious food ingredients (Ke and Pang, 2026). To date, soybean protein has been the most extensively studied plant protein for emulsion-templated oleogel fabrication. For example, soybean protein-based oleogels incorporating mango kernel fat have demonstrated favorable digestive stability and bioactive delivery capacity (Song et al., 2025). Composite systems of soybean protein isolate with chitosan or xanthan gum have yielded oleogels with compact microstructures and enhanced rheological properties, showing promise as solid fat replacers in frozen food and dietary applications (Zhu et al., 2024, Zhu et al., 2024; Sinha et al., 2024). Despite these advances, soybean protein possesses several intrinsic limitations that constrain its broader application. Its relatively low interfacial activity generally necessitates incorporating polysaccharides or other biopolymers to reinforce the gel network, thereby adding formulation complexity. Moreover, the characteristic beany flavor and allergenicity of soybean protein remain persistent barriers to consumer acceptance in diverse food matrices. Other plant proteins, such as zein, have also been explored; however, the poor aqueous solubility of zein requires the addition of exogenous surfactants (*e.g*., tea saponin) (Liu et al., 2024, Liu et al., 2024). Overall, existing studies have shown the feasibility of using plant proteins to structure liquid oils; however, these systems mainly rely on commonly studied proteins, and their oleogel properties depend heavily on protein concentration and processing conditions. In comparison, the potential of proteins derived from agricultural by-products remains relatively underexplored.

Corn silk, the stigma and style of maize, is an agricultural by-product generated in large quantities during corn processing, yet it remains largely discarded or confined to traditional herbal applications. Recent studies have revealed that corn silk is a rich repository of bioactive compounds, including polysaccharides and polyphenols with demonstrated antioxidant activity, as well as protein fractions (albumin, glutelin, and globulin) that exhibit inherent emulsifying capacity (Orellana-Palacios et al., 2025). These attributes position corn silk protein as a compelling candidate for emulsion-based food applications. However, native plant proteins often possess compact structures that may limit their interfacial and functional properties. Although researchers have recently characterized the composition, structural characteristics, and techno-functional properties of corn silk protein, little is known about how processing conditions, such as pH treatment, affect its structure and functionality. More importantly, translating these functional properties into food material systems and potential applications remains largely unexplored. In particular, whether the emulsifying properties of corn silk protein can be used to construct emulsion-templated oleogels remains unclear.

Herein, corn silk protein was extracted by alkaline solubilization-acid precipitation, and the effects of pH treatment (pH 3, 5, 7, 9, and 11) on its structural and functional properties were systematically investigated. Its potential as a natural emulsifier for constructing emulsion-templated oleogels was further evaluated. We hypothesized that pH treatment could alter the structure and functional properties of corn silk protein, thereby affecting its emulsifying performance, emulsion-template formation, and subsequent oleogel formation. Accordingly, this study provides preliminary insights into the potential relationships among pH-dependent structural changes, emulsifying properties, emulsion-template formation, and oleogel formation. By extending previous research beyond the basic characterization of corn silk protein toward the modulation and application of its functional properties, this work also provides a potential approach for the value-added utilization of corn silk protein in structured food systems.

## 2 Materials and methods

### 2.1 Materials

Corn silk was sourced from fresh maize collected in Nanning, Guangxi Province, China, and sun-dried before use. Corn oil was purchased from NTUC FairPrice Co-operative Ltd. (Singapore). All chemicals and reagents used were of analytical grade, and deionized water was used throughout the experiments.

### 2.2 Preparation of samples

#### 2.2.1 Extraction of corn silk protein

Corn silk protein was extracted using the alkaline extraction-acid precipitation method. Briefly, dried corn silk was ground and sieved through a 40-mesh screen. The resulting powder was dispersed in 0.1 mol/L NaOH at a solid-to-liquid ratio of 1:20 (w/v) and stirred at 35 °C for 2 h. The suspension was then centrifuged at 3860 × g for 30 min (CL5R, Xiangyi Laboratory Instrument Development Co., Ltd., Changsha, China), and the supernatant was collected. Subsequently, the pH of the supernatant was adjusted to 4.0 with 1 mol/L HCl to induce protein precipitation. After incubation for 2 h, the mixture was centrifuged at 3860 × g for 30 min. The precipitate was collected and redispersed in deionized water. Then, the protein solution was dialyzed (molecular weight cut-off: 7000–14,000 Da) against deionized water for 48 h and subsequently freeze-dried for further use.

#### 2.2.2 pH modification of corn silk protein

Corn silk protein solutions (10 mg/mL, nominal concentration) were adjusted to pH 3, 5, 7, 9, and 11 using 1 mol/L HCl or 1 mol/L NaOH and then stirred for 2 h at room temperature. The samples were denoted as C3, C5, C7, C9, and C11, respectively. The corn silk protein solution without pH adjustment was used as the control (C, pH 6.55).

#### 2.2.3 Preparation of corn silk protein-based oleogels

Corn silk protein-stabilized emulsions were prepared at water-to-oil volume ratios of 5/5, 4/6, 3/7, 2/8, and 1/9 using an IKA T10 homogenizer (IKA, Staufen, Germany) at 28,800 rpm for 2 min, and denoted as E5, E6, E7, E8, and E9, respectively. The aqueous phase consisted of CSP solutions at pH 11 with a nominal concentration of 10 mg/mL (Section 2.2.2, C11), while corn oil was used as the oil phase. The emulsions were then dried in an oven at 40 °C for 24 h to obtain oleogels, denoted as O5, O6, O7, O8, and O9, corresponding to their respective water-to-oil volume ratios. Corn oil underwent the same thermal treatment (40 °C, 24 h) and served as the control (O).

### 2.3 Structural characterization of corn silk protein

#### 2.3.1 Free sulfhydryl group content

The free sulfhydryl (-SH) content was determined using Ellman's reagent (5,5′-dithiobis (2-nitrobenzoic acid), DTNB). The Tris-glycine (Tris-Gly) buffer was prepared by dissolving Tris (10.4 g), glycine (6.9 g), EDTA (1.2 g), and urea (480 g) in 1 L of deionized water. Ellman's reagent was prepared by dissolving DTNB (0.2 g) in the Tris-Gly buffer. For measurement, the protein solution (1.25 mL) was mixed with Tris-Gly buffer (3.75 mL) and Ellman's reagent (50 μL), followed by incubation at 40 °C for 25 min. The absorbance was measured at 412 nm. The free sulfhydryl content was calculated using Eq. (1):

(1) −SH(μmolg)=73.53×A412×DC

Where A_412_ is the difference value between the absorbance of the sample with Ellman reagent and the sample without Ellman reagent. D represents the dilution factor of the sample, and C is the protein concentration of the sample (mg/mL).

#### 2.3.2 Fourier Transform Infrared Spectroscopy (FTIR)

The FTIR spectra of the samples were recorded using an ATR-FTIR spectrometer (Agilent Cary 630, Agilent Technologies, USA) over a wavenumber range of 4000-650 cm^−1^ with 32 scans at a resolution of 4 cm^−1^. The amide I band (1700-1600 cm^−1^) was subjected to Fourier self-deconvolution, followed by Gaussian peak fitting using PeakFit software (Systat Software Inc., San Jose, CA, USA) to determine the secondary structure content of the protein. The fitted peaks were assigned to α-helix (1650-1660 cm^−1^), β-sheet (1610-1640 cm^−1^), β-turn (1660-1700 cm^−1^), and random coil (1640-1650 cm^−1^). The relative content of each secondary structure was calculated from the corresponding peak area as a percentage of the total fitted peak area in the amide I region.

#### 2.3.3 Scanning Electron Microscopy (SEM)

The corn silk protein solution was deposited onto a silicon wafer and dried. The dried samples were then mounted onto conductive adhesive tape and sputter-coated with gold. Microstructures were observed using a field-emission scanning electron microscope (FESEM, JSM-6701F, JEOL Ltd., Japan) at an accelerating voltage of 5 kV.

### 2.4 Functional properties of corn silk protein

#### 2.4.1 Solubility

The samples were centrifuged at 10,000 × g for 20 min. The soluble protein content was determined using a BCA kit (Beyotime Biotechnology, China). The supernatant (20 μL) was mixed with BCA reagent (200 μL) and incubated for 2 h at 25 °C. The absorbance was measured at 562 nm using a Biotek Synergy HTX Multi-mode Microplate Reader (BioTek Instruments, Inc., Winooski, VT, USA). A standard curve was established using bovine serum albumin (BSA) at concentrations of 0.005, 0.01, 0.025, 0.05, 0.10, 0.15, and 0.20 mg/mL. The soluble protein content (mg/mL) was calculated by substituting the value into the standard curve (y = 0.9372x + 0.0866, R^2^ = 0.9978). The solubility was calculated using Eq. (2):

(2) $$\text{Solubility}\;\left(\%\right)=\frac{\text{soluble}\;\text{protein}\;\text{content}}{\text{total}\;\text{protein}\;\text{content}\;\text{in}\;\text{sample}}\times 100$$

#### 2.4.2 Emulsifying property

The protein solution was mixed with corn oil at a volume ratio of 3:1 and homogenized for 2 min at 28,800 rpm using an IKA T10 homogenizer (IKA, Staufen, Germany). The homogenized sample (0 min and 10 min, 50 μL) was mixed with a 0.1% SDS (Sodium dodecyl sulfate) solution. The absorbance was measured at 500 nm (A_500_). The emulsifying activity index (EAI) and emulsifying stability index (ESI) were calculated using Eqs. (3), (4):

(3) EAI(m2g)=2×2.303×A0×N10000×θ×L×C

(4) $$\text{ESI}\;\left(\min\right)=\frac{A_{0}\times 10}{A_{0}-A_{10}}$$

Where N represents the dilution factor, θ is the volume ratio of the oil phase (0.25), L is the thickness of the cuvette (1 cm), C indicates the concentration of the protein solution prior to emulsification (g/mL). A_0_ and A_10_ are the absorbance values at 500 nm measured at 0 and 10 min, respectively.

#### 2.4.3 Thermal stability

Thermal stability was evaluated using differential scanning calorimetry (DSC 25, TA Instruments, USA). Freeze-dried sample powder (5 mg) was placed in an aluminum crucible and heated from 30 to 250 °C at a rate of 10 °C/min under a nitrogen flow of 30 mL/min.

### 2.5 Zeta-potential and particle size

The zeta-potential and particle size of the corn silk protein-based emulsion were measured using a Brookhaven Zeta-potential and Nano-Particle Sizer (Brookhaven Instruments Corporation, USA). Emulsion samples were diluted 10-fold with DI water and measured in triplicate at 25 °C, with refractive indices of 1.467 and 1.330 for corn oil and DI water, respectively.

### 2.6 Confocal Laser Scanning Microscopy (CLSM)

The oil phase was stained with Nile red (1 mg/mL), while the protein phase was stained with Nile blue (1 mg/mL). Nile red was excited at 488 nm, and Nile blue at 633 nm. Images were acquired using a 40× objective lens on a laser scanning confocal microscope (Olympus FV3000, Japan).

### 2.7 Properties of corn silk protein-based oleogels

#### 2.7.1 Texture analysis (Hardness measurement)

The oleogel samples were placed in a cylindrical container with a diameter of 35 mm. The hardness was measured using a Stable Micro Systems Texture Analyzer (TA-XT2i, SMS, UK) equipped with an SMA P/20 cylindrical probe. The test parameters were set as follows: pre-test speed of 2.0 mm/s, test speed of 1.0 mm/s, post-test speed of 1.0 mm/s. The trigger force was set to 5 g, and the compression distance was set to 50% of the original sample height.

#### 2.7.2 Rheological analysis

The rheological properties of the oleogels were evaluated using a Rheometer MCR 102 (Anton Paar, Austria) with a parallel-plate (40 mm diameter) and a gap of 1000 μm. All measurements were conducted at 25 °C. The strain sweep was performed in the range of 0.01-100% strain at a constant frequency of 1.0 Hz. The oscillatory frequency sweep tests were carried out logarithmically over a frequency range of 1-100 Hz at a constant strain of 0.1%. The steady shear viscosity of the oleogels was determined by measuring the flow behavior across a shear range of 0.1-100 s^−1^.

#### 2.7.3 Oil Holding capacity

Corn silk protein-based oleogels (m, 1 g) were taken into a 15 mL centrifuge tube. The mass of the tube containing the oleogel was recorded as m_1_. Then, the sample was centrifuged at 12,800 × g for 15 min using a centrifuge (Eppendorf 5810 R, Hamburg, Germany). After centrifugation, the released oil was decanted, and residual surface oil was removed with filter paper. The mass of the tube containing the remaining oleogels was then recorded as m_2_. The oil holding capacity (OHC) was calculated using Eq. (5):

(5) $$\text{Oil}\;\text{Holding}\;\text{Capacity}\;\left(\text{OHC},\%\right)=\frac{m‐\left(m_{1}‐m_{2}\right)}{m}\times 100$$

#### 2.7.4 Oxidation stability

The oleogel samples were stored at 25 °C, and the primary and secondary oxidation products were analyzed on days 1, 3, 5, and 7. Liquid corn oil subjected to the same 40 °C for 24 h treatment was used as the control.

##### 2.7.4.1 Primary oxidation products

The oleogel samples (0.3 g) were mixed with 1.5 mL of isooctane/isopropanol (3:2, v/v) solution and centrifuged at 1160 × g for 5 min. The supernatant (200 μL) was mixed with 2.8 mL of methanol/n-butanol (2:1, v/v) solution, 15 μL of ammonium thiocyanate solution (3.94 mol/L), and 15 μL of FeCl_2_ (0.072 mol/L, prepared from the reaction between BaCl_2_ and FeSO_4_). The mixture was incubated in the dark at 25 °C, for 20 min. The absorbance was measured at 510 nm, and the peroxide value (POV) was calculated using a calibration curve constructed with hydrogen peroxide standards.

##### 2.7.4.2 Secondary oxidation products

The oleogel samples (0.3 g) were mixed with thiobarbituric acid (TBA, 2 mL) and centrifuged at 12,800 × g for 15 min. The absorbance of the supernatant was measured at 532 nm. The TBA reagent (1 L) was prepared by dissolving 15 g trichloroacetic acid, and 0.375 g thiobarbituric acid in water containing100 mL HCl (4 mol/L). A calibration curve was constructed using 1,1,3,3-tetraethoxypropane as the standard, and the concentration of thiobarbituric acid reactive substances (TBARS) was calculated accordingly.

### 2.8 Statistical analysis

All measurements were performed in triplicate. One-way analysis of variance (ANOVA) and Duncan's multiple comparisons were used for evaluating statistical significance, with *p < 0.05* considered statistically significant. For the oxidation stability data, one-way ANOVA was performed separately at each storage time point to compare the oleogel formulations on the same storage day. Statistical analyses were performed using SPSS 25.0 (USAIBM Corp., Armonk, NY, USA). Results are presented as mean ± standard deviation (SD). All data were plotted using Origin 2025 (OriginLab Corporation, USA).

## 3 Results and discussion

### 3.1 Structural characterization

#### 3.1.1 Free sulfhydryl group content

Free sulfhydryl (-SH) groups are important indicators of protein conformational changes, as they reflect the exposure of cysteine residues and the status of disulfide bonds. As shown in Fig. 1A, under acidic conditions (C3 and C5), the free sulfhydryl content showed an increase (2.98 and 4.44 μmol/g, respectively) compared with the control CSP (C, 2.33 μmol/g). This increase may be primarily attributed to partial unfolding of the protein structure, which may expose previously buried sulfhydryl groups. Since acidic conditions alone are insufficient to cleave disulfide bonds, the observed increase in free sulfhydryl groups is more likely attributable to enhanced accessibility of previously buried sulfhydryl groups rather than disulfide bond cleavage. Notably, the free sulfhydryl content of C3 was lower than that of C5, possibly because pH 3 is closer to the protein's isoelectric point, where reduced electrostatic repulsion promotes aggregation and partial burial of sulfhydryl groups (Liu et al., 2025). At pH 9 (C9), the free sulfhydryl content further increased significantly to 5.64 μmol/g, indicating more pronounced structural unfolding and greater availability of reactive thiol groups (Huang et al., 2025). In contrast, a decrease in free sulfhydryl content was observed at pH 11 (C11, 1.07 μmol/g). Although alkaline-induced unfolding may expose previously buried sulfhydryl groups, the highly alkaline environment may also promote thiol oxidation and thiol-disulfide exchange reactions, potentially contributing to the formation of disulfide bonds. Therefore, despite potentially more extensive protein unfolding at pH 11, thiol oxidation and thiol-disulfide exchange may contribute to lower measurable free sulfhydryl content (Xie et al., 2026).

**Fig. 1:**
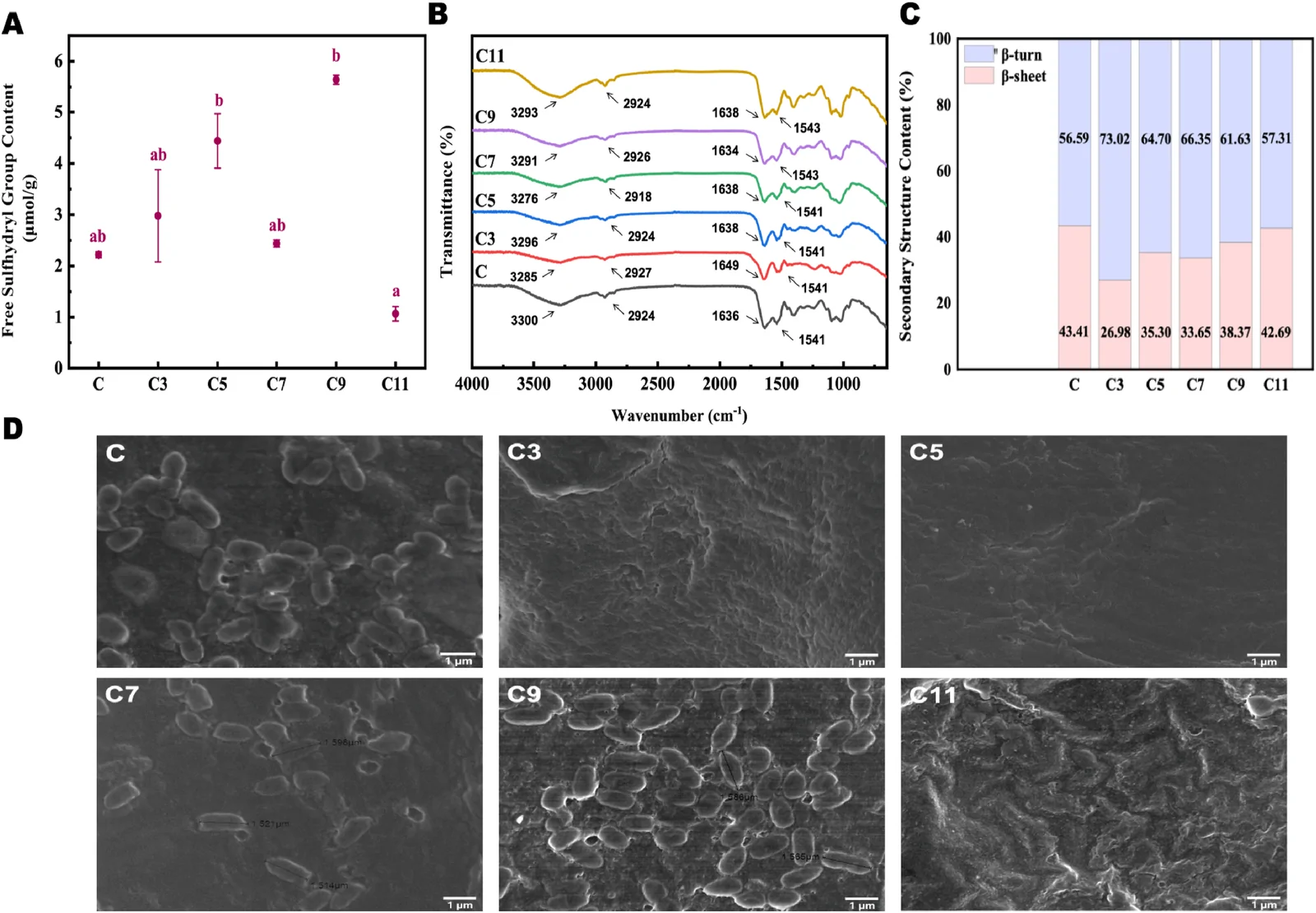
Characterization of corn silk protein (CSP) treated at different pH values (3, 5, 7, 9, and 11). (A) Free sulfhydryl content. (B) Fourier transform infrared (FTIR) spectra. (C) Secondary structure composition determined from FTIR deconvolution. (D) Scanning electron microscopy (SEM) images showing the microstructural evolution of CSP following pH treatment. CSP without pH treatment (pH 6.55) was used as the control (C).

#### 3.1.2 Fourier Transform Infrared Spectroscopy

FTIR was used to further investigate pH-induced structural changes in corn silk protein, with particular focus on secondary structure and hydrogen-bonding interactions (Fig. 1B and C). The amide A band (3500-3200 cm^−1^), corresponding to N-H and O-H stretching vibrations, shifted toward lower wavenumbers after both acidic and alkaline treatments (Fig. 1B). Such red shifts indicate changes in hydrogen bonding environments, which may arise from alterations in protein-protein interactions following structural rearrangement. Amide B (3000-2800 cm^−1^) represents the C-H stretching region. Compared with C, C5 and C11 showed no significant shifts, whereas C7 exhibited a red shift, possibly due to a partial rearrangement of the protein structure that altered the microenvironment surrounding the hydrophobic side chains (Koamornsup et al., 2026). In contrast, C9 showed a blue shift, indicating reduced intermolecular interactions, suggesting a more flexible, less aggregated state. Note that the peak intensity of C11 in amide B increased, which may be associated with structural unfolding and greater exposure of hydrophobic groups. In contrast, the peak intensity of C3 decreased, possibly due to structural disruption and subsequent aggregation that may bury hydrophobic groups. Amide II (1580-1510 cm^−1^) represents N-H bending coupled with C-N stretching vibrations. No significant shift was observed in C3, C5, and C7 compared to the control (C). However, the blue shift of the amide II band under alkaline conditions (C9 and C11) suggests alterations in the local environment of the peptide backbone, possibly due to partial protein unfolding.

To further quantify secondary structural changes, the amide I band (1700-1600 cm^−1^) was analyzed by spectral deconvolution. Generally, secondary structures in proteins, including α-helix, β-sheet, β-turn, and random coil, could be assessed based on C=O stretching vibration (Sarker et al., 2026). The results of this study indicate that β-sheet and β-turn structures dominate in corn silk protein (Fig. 1C), consistent with a previous study in which β-structures were identified as the predominant secondary structural components (Orellana-Palacios et al., 2025). Upon pH treatment, a decrease in β-sheet content and an increase in β-turn structures were observed, indicating a rearrangement of the protein secondary structure, possibly associated with partial unfolding or conformational changes (Liu et al., 2025). In summary, FTIR analysis suggests that pH treatment alters the structure of corn silk protein, as reflected by changes in its secondary structural components. These structural changes be favorable for subsequent protein adsorption at the oil-water interface.

#### 3.1.3 Scanning Electron microscopy (SEM)

Various microscopic structures of corn silk protein were observed at different pH values, indicating that pH treatment markedly influenced the structural characteristics of corn silk protein, as shown in Fig. 1D. The control CSP (C) exhibited relatively uniform particles with ellipsoidal to sub-spherical morphology and smooth surfaces, with an average diameter of approximately 1.5-1.6 μm. However, partial particle aggregation was observed, suggesting possible intermolecular interactions among protein molecules, such as electrostatic interactions. The structure of corn silk protein was significantly damaged after treatment with a strong acid (pH 3), as evidenced by the loss of its spherical structure and the formation of aggregates. This behavior is consistent with reduced electrostatic repulsion near the isoelectric point, which promotes protein-protein association and aggregation (Liu et al., 2025). In C5, the native structure of corn silk protein was disrupted, accompanied by protein aggregation, likely due to mild acid treatment that altered the original protein conformation and induced aggregation and/or structural rearrangement. In contrast, at neutral pH (C7), part of the corn silk protein still retained its elliptical-spherical structure, with relatively uniform diameters of approximately 1.5 μm and well-defined edges, indicating that the native-like structure was largely preserved under this condition. Under the moderately alkaline condition (C9), the protein particles largely retained their original ellipsoidal morphology with well-defined edges. Compared with the control, the degree of intermolecular aggregation was reduced, resulting in a more extended and continuous conformation. This suggests that mild alkaline treatment promoted partial unfolding and may increase molecular flexibility. In contrast, under the strongly alkaline condition (C11), the characteristic particle morphology was no longer observed. The disappearance of the well-defined ellipsoidal structures and the presence of irregular, fragmented features suggest extensive structural disruption under these conditions. This structural alteration may enhance protein interfacial activity and form a relatively stable interfacial film at the oil-water interface.

### 3.2 Functional properties

#### 3.2.1 Solubility

Protein solubility is a fundamental functional property that reflects the balance between protein-protein and protein-water interactions and plays a critical role in determining interfacial behavior. C9 and C11 showed slightly higher solubility values than C (69.7%), reaching 72.3% and 75.8%, respectively, as shown in Fig. 2A. This improvement may be associated with enhanced electrostatic repulsion and structural unfolding at elevated pH, which may reduce protein aggregation and promote dispersion. In particular, the increased net negative charge at alkaline pH may enhance protein hydration and interactions with the aqueous phase. The slightly higher solubility observed for C11 compared with C9 is likely due to more extensive structural rearrangement (Fig. 1D), potentially resulting in greater exposure of hydrophilic groups.

**Fig. 2:**
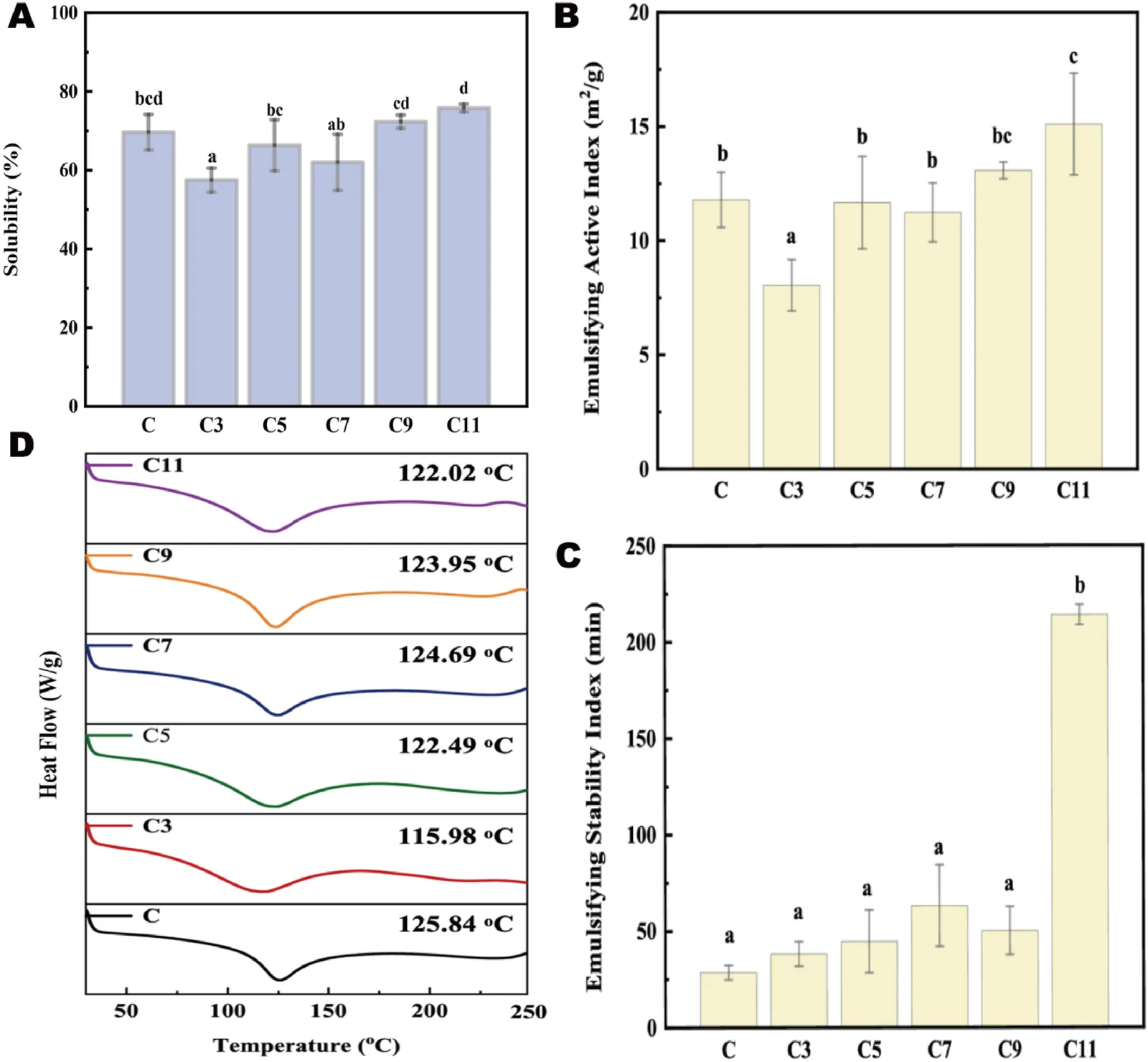
Functional properties of corn silk protein (CSP) treated at different pH values (3, 5, 7, 9, and 11). (A) Solubility. (B) Emulsifying activity index (EAI). (C) Emulsifying stability index (ESI). (D) Thermal stability. Different lowercase letters indicate statistically significant differences *(p < 0.05)*.

The solubility of corn silk protein at neutral pH (C7) remained relatively low (62.0%), which may be associated with protein aggregation at this pH (Li et al., 2026). The solubility of corn silk protein decreased under acidic conditions, with C3 showing the lowest value (57.5%). This behavior is consistent with the proximity to the isoelectric point (approximately pH 3.5), where reduced net charge minimizes electrostatic repulsion and promotes protein aggregation (Orellana-Palacios et al., 2025). The formation of compact aggregates limits protein-water interactions, thereby reducing solubility. At pH 5 (C5), the solubility (66.3%) was higher than at pH 3, attributable to a partial recovery of net charge, which increased electrostatic repulsion and improved dispersion. Of note, pH-induced changes in solubility are closely associated with alterations in protein charge, conformation, and aggregation behavior. Enhanced solubility under alkaline conditions reflects improved dispersion and molecular flexibility, which are expected to facilitate protein adsorption and rearrangement at oil-water interfaces, thereby enhancing emulsifying properties.

#### 3.2.2 Emulsifying properties

Protein emulsifying properties are strongly dependent on their structural characteristics and ability to adsorb at the oil-water interface. Previous studies have shown that pH treatment can modify protein conformation and intermolecular interactions, thereby influencing emulsifying activity and stability. As shown in Fig. 2B, C3 exhibited significantly decreased EAI compared with C. At a pH near the isoelectric point of corn silk protein (approximately pH 3.5), the net charge of the protein is minimized, leading to reduced electrostatic repulsion and enhanced aggregation. The formation of large aggregates limits protein solubility and mobility (Fig. 2A), thereby restricting diffusion to the interface and reducing effective interfacial coverage (Li et al., 2026). In contrast, C11 exhibited the highest EAI (15.11 m^2^/g) and ESI (214.11 min) (Fig. 2B and C). This improvement can be attributed to pH treatment, which may induce structural unfolding and increased molecular dispersion, enhancing protein mobility in the aqueous phase. In addition, the structural rearrangement may promote a more favorable balance between hydrophobic and hydrophilic groups, potentially facilitating protein adsorption at the oil-water interface. These factors may enable protein molecules to rapidly adsorb and reorganize at the interface, forming a more continuous and mechanically stable interfacial film, which contributes to improved emulsifying activity and stability (Yang et al., 2025). Compared with C11, C9 showed lower EAI (13.08 m^2^/g) and ESI (50.23 min). This may be attributed to the incomplete unfolding of corn silk protein or to structural rearrangement (Fig. 1D), resulting in aggregate formation that limited the formation of a dense interfacial layer and prevented complete coverage of oil droplets. Additionally, the EAI values at pH 5 and 7 were higher than those at pH 3, likely because these pH conditions are further from the isoelectric point of corn silk protein, reducing protein aggregation, and the increased solubility (Fig. 2A) enhanced the interfacial dispersion at the oil-water interface.

#### 3.2.3 Thermal stability

Differential Scanning Calorimetry (DSC) was used to determine the thermal denaturation temperature (T_d_) of corn silk protein, which reflects its thermal stability. As shown in Fig. 2D, T_d_ of the pH-treated corn silk proteins decreased to varying extents compared with that of control CSP (C, 125.84 °C). This indicates that pH treatment modified the protein structure and reduced its structural stability, thereby requiring less thermal energy to disrupt the non-covalent interactions that maintain the protein structure, which is consistent with pH-induced conformational changes (Sharma et al., 2026). The thermal stability under neutral (C7, 124.69 °C) and mild alkaline conditions (C9, 123.95 °C) is comparable to that of C. This may be because the protein's conformation is relatively close to its natural state, which is consistent with the results of SEM (Fig. 1D). The denaturation temperature of corn silk protein decreased under both strong acidic (C3, 115.98 °C) and strong alkaline conditions (C11, 122.02 °C), indicating reduced thermal stability. This is because the thermal stability of proteins is related to their compact hydrophobic core structure (Aider et al., 2025). Specifically, treatment with strong acids and strong alkalis may induce significant protein structural unfolding, accompanied by the exposure of previously buried hydrophobic groups. These structural changes may weaken hydrophobic interactions between protein molecules, reducing the energy required for thermal transitions and thereby decreasing thermal stability. Overall, the thermal stability of proteins is more readily compromised under strong acid and strong alkaline conditions, whereas it is better maintained under neutral and mild alkaline conditions.

In summary, mild acid and alkaline treatments (pH 5 and 9) increased the exposure of free sulfhydryl groups, possibly due to partial unfolding or structural rearrangement without extensive denaturation. Under neutral conditions (pH 7), CSP largely retained its native-like structure, with only limited structural rearrangement. In contrast, strong acid treatment (pH 3) resulted in pronounced structural disruption and aggregation. Strong alkaline treatment (pH 11) was associated with more pronounced structural changes, which may involve protein unfolding and greater exposure of previously buried hydrophobic groups. These structural changes may facilitate protein adsorption at the oil-water interface and contribute to improved solubility and emulsifying properties observed at pH 11.

### 3.3 Zeta-potential and particle size of emulsions

Given its superior interfacial performance, C11 was selected to fabricate emulsions at different water-to-oil volume ratios (E5-E9) as templates for oleogel preparation. The appearances of the corn silk protein-stabilized emulsions are shown in Fig. S1. E5, E6, and E7 were homogeneous and uniformly turbid. In contrast, E8 and E9 exhibited a free-oil layer, indicating incomplete incorporation of the oil phase into the emulsions. This was likely associated with the substantially increased oil-water interfacial area at oil fractions of 80% and 90%, which may have exceeded the interfacial coverage capacity of the available corn silk protein.

The zeta-potential of the emulsion reflects the surface charge of the droplets and is closely associated with emulsion stability. E7 exhibited the highest absolute value (49.51 mV), indicating a high energy barrier between emulsion droplets, which effectively prevents droplet flocculation and demonstrates excellent emulsion stability (J. Zhu et al., 2024). The absolute values of the zeta-potential of E5 (26.87 mV) and E6 (39.14 mV) were lower than that of E7. This may be caused by excessive amounts of unadsorbed soluble protein and nonprotein components in the systems, which may alter the ionic environment surrounding the droplets through electrostatic screening, thereby lowering the absolute value of the zeta-potential (H. Li et al., 2025). The absolute values of the zeta-potential of E8 (49.02 mV) and E9 (40.15 mV) were lower than that of E7. Additionally, the absolute value of the zeta-potential of E9 was significantly reduced compared to E7 and E8. This may be because when the volume of the oil phase became too large, there was insufficient corn silk protein to form a stable interfacial film on the surface of the oil droplets (Wang et al., 2023).

The particle size of the emulsion increased as the oil phase volume increased, as shown in Table S1. At lower oil volume fractions, the higher protein-to-oil ratio provided sufficient protein molecules to rapidly adsorb onto oil droplets, resulting in the formation of a more complete and stable interfacial film. This effectively suppressed droplet flocculation and coalescence, thereby forming emulsions with smaller particle sizes (Wang et al., 2023). As the volume of the oil phase increased, insufficient protein adsorption onto the oil droplet surface led to flocculation and coalescence, particularly in E8 and E9.

### 3.4 Confocal Laser scanning Microscopy

The microscopic structure of the oleogels was observed by confocal laser scanning microscopy (CLSM) and is shown in Fig. 3A, with the oil phase stained red and the protein phase stained green. In O5, it can be observed that proteins form a continuous yet irregular, scaffold-like three-dimensional network capable of entrapping liquid oil, despite localized protein aggregation and clustering. In the merged image, the overlap of green and red signals (yellow areas) indicates that the oil phase was embedded within the protein network, although its uneven distribution, appearing as scattered points or blocks, suggests localized aggregation. Compared with O5, O6 exhibited a more continuous and porous protein scaffold capable of entrapping the oil phase, although pronounced protein aggregation and localized oil-droplet clustering remained, indicating an uneven network structure. O7 exhibited a more continuous and structurally integrated protein network than O5 and O6, contributing to a more uniform distribution of the oil phase, although slight oil droplet aggregation was still present. The three-dimensional network structure of corn silk protein was not observed in O8 and O9. At excessively high oil-phase ratios, the amount of protein available relative to the oil phase decreased, which may limit the amount of protein available to stabilize the increasing oil-water interfacial area. Consequently, droplet coalescence and aggregation occurred, as evidenced by the significantly larger particles observed for E8 and E9 (Table S1). The resulting unstable emulsions were more prone to phase separation, preventing the formation of a continuous network during drying and ultimately hindering oleogel formation (Wang et al., 2023). The appearance of the oleogels showed that O5, O6, and O7 exhibited a soft solid-like state, whereas O8 and O9 remained flowable and displayed liquid-like characteristics (Fig. 3B). Notably, O7 maintained its shape without flowing upon inversion among all samples, indirectly indicating the formation of a more stable network structure compared with the other groups. Therefore, subsequent oleogel characterizations were conducted for O5, O6, and O7.

**Fig. 3:**
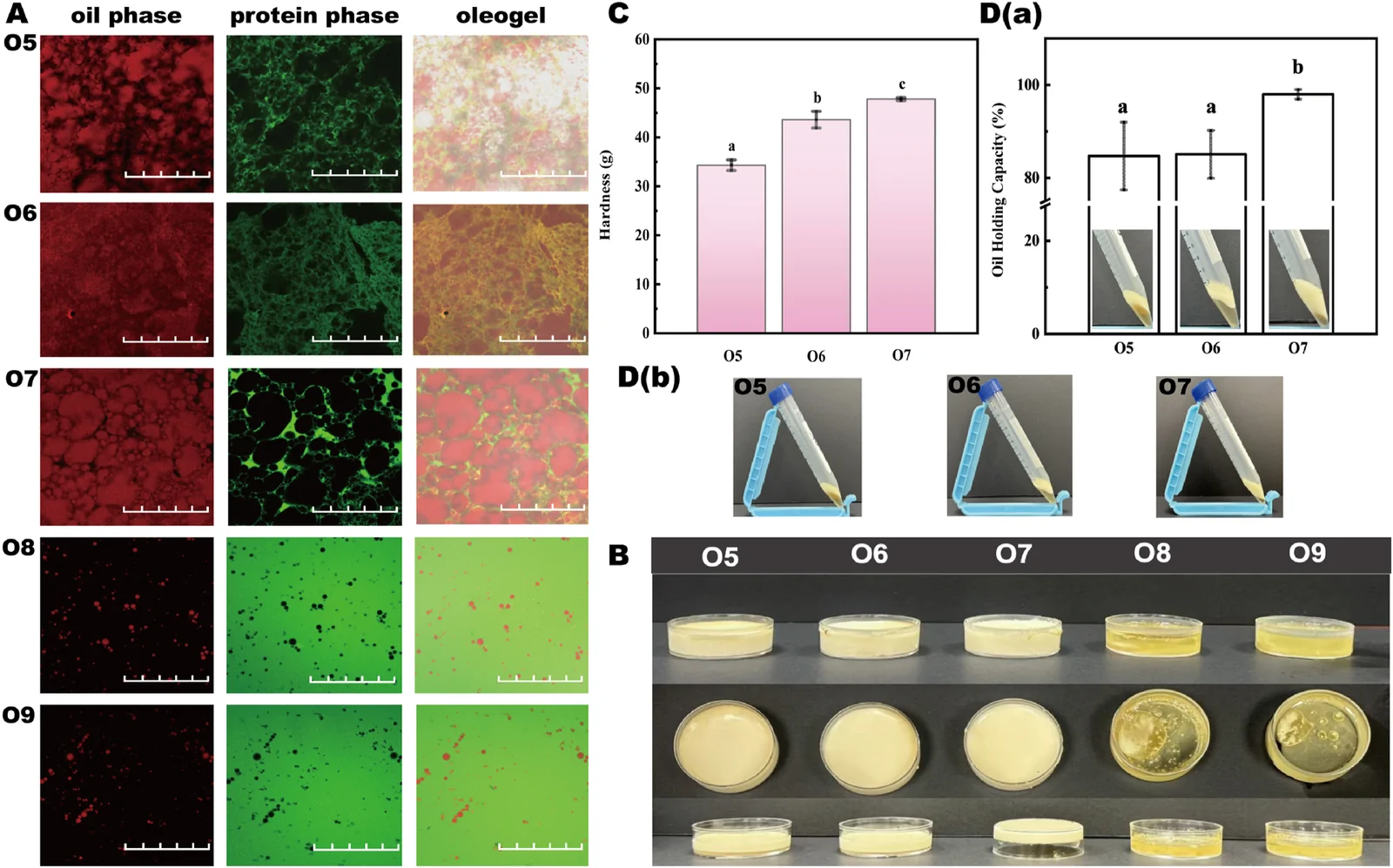
Characterization of corn silk protein-based oleogels prepared from emulsions with different water-to-oil volume ratios (5/5, 4/6, 3/7, 2/8, and 1/9), denoted as O5, O6, O7, O8, and O9, respectively. (A) Confocal laser scanning microscopy (CLSM) images (scale bar = 50 μm), where red and green represent the oil and protein phases, respectively. (B) Macroscopic appearance and inverted-vial test of the oleogels. (C) Hardness. (D) Oil-holding capacity (a) and appearance after centrifugation (b). Different lowercase letters indicate statistically significant differences *(p < 0.05)*.

### 3.5 Hardness

Hardness not only indicates the oleogel's ability to resist deformation under external pressure but also indirectly reveals its structural properties (Zhao et al., 2025, Zhao et al., 2025). In plant protein-based oleogels, proteins act as scaffolding agents that facilitate the formation of a network and are essential to the hardness of the oleogels (Feng et al., 2025). The hardness of the oleogel increased with increasing oil phase volume, and O7 exhibited the highest hardness among O5-O7, as shown in Fig. 3C. The higher hardness of O7 may be attributed to the formation of a denser protein network that restricted the mobility of the continuous oil phase, whereas the more porous structure and larger pore size observed in O5 and O6 provided weaker structural support, resulting in lower hardness, consistent with the CLSM observation (Fig. 3A).

### 3.6 Oil holding capacity

Oil-holding capacity (OHC) reflects both the structural stability of oleogels and the ability of the protein network to immobilize the oil phase (Liu et al., 2024). As shown in Fig. 3D, O7 exhibited the highest OHC (98.00%), significantly higher than those of O5 (84.73%) and O6 (85.08%). This may be attributed to the formation of a more organized and mechanically robust protein network at an oil-phase volume of 70%, where corn silk protein formed a more uniform interfacial layer at the oil-water interface, thereby stabilizing the emulsion template and reducing oil leakage (Luo et al., 2025). This finding is consistent with previous studies showing that oleogels with denser and more robust gel networks, characterized by higher hardness and elasticity, generally exhibit greater oil-holding capacity, which corresponds to the superior hardness observed in O7 (Mert and Vilgis, 2021).

### 3.7 Rheological properties of corn silk protein-based oleogels

#### 3.7.1 Viscosity

The viscosity of all oleogels decreased as the shear rate increased (Fig. 4A). This indicates that all the samples exhibited a shear-thinning behavior. This behavior is primarily due to the progressive disruption of the protein network under shear, accompanied by the breakdown and release of entrapped oil droplets, thereby reducing the flow resistance and apparent viscosity of gels (Yang et al., 2024). O7 exhibited the highest viscosity, suggesting that this water-to-oil ratio may provide a more favorable balance between interfacial coverage and protein network formation, potentially facilitating the development of a three-dimensional protein network capable of entrapping oil and resisting deformation. Similarly, in an emulsion-templated oleogel system made with mango kernel fat, the highest viscosity was observed when the volume ratio of soybean protein isolate solution to oil phase was 3:7 (Song et al., 2025). In contrast, O5 exhibited a lower viscosity, which was mainly attributed to its loose and discontinuous protein network. These results suggest that oleogel formation is composition dependent, with both insufficient and excessive oil loading compromising network integrity, whereas an optimal oil fraction promotes the formation of a continuous and robust three-dimensional network.

**Fig. 4:**
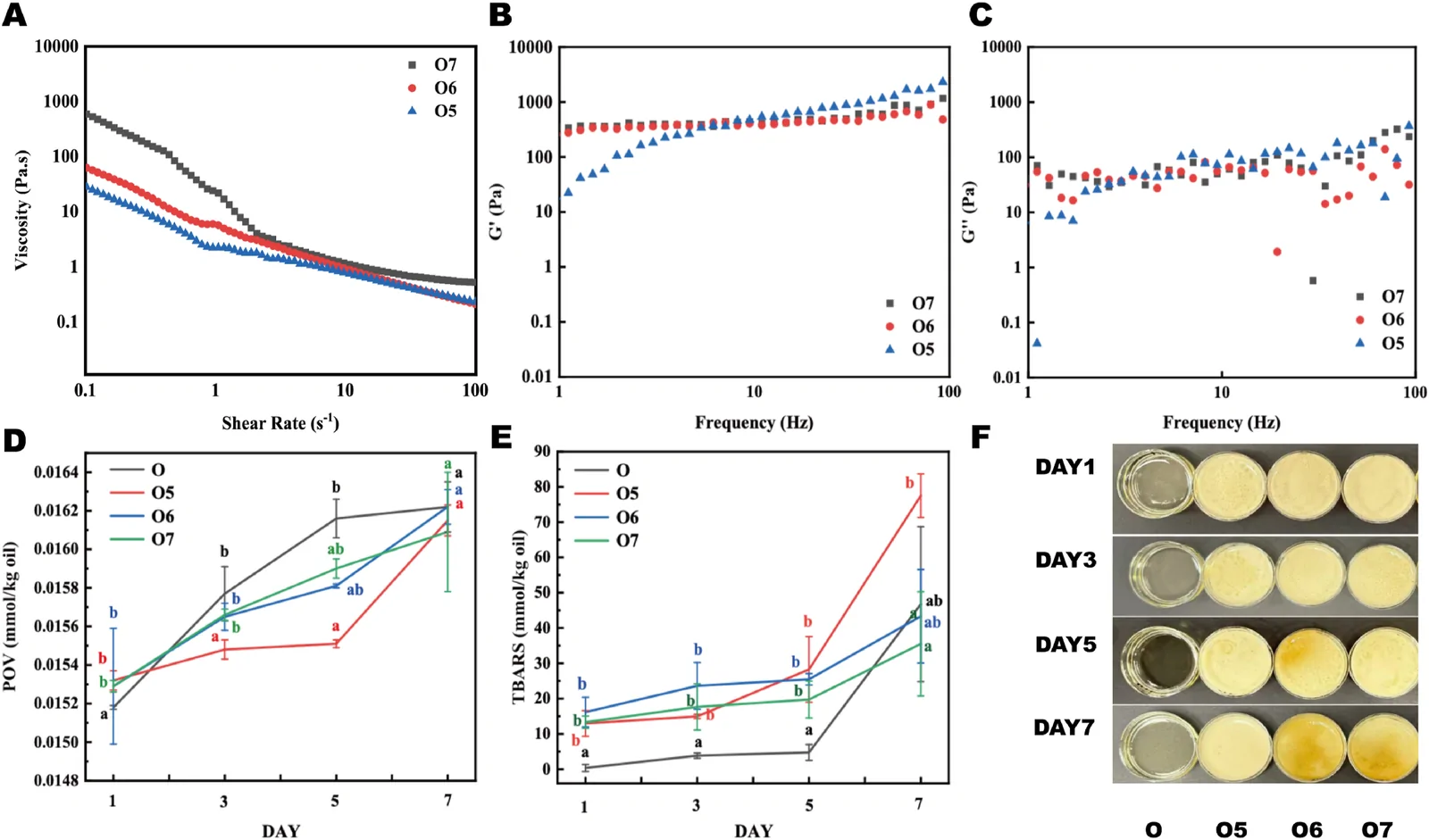
Rheological behavior and oxidative stability of corn silk protein-based oleogels prepared with water-to-oil volume ratios of 5/5 (O5), 4/6 (O6), and 3/7 (O7). (A) Apparent viscosity. (B, C) Frequency-dependent storage modulus (G′) and loss modulus (G″). (D) Peroxide value (POV). (E) Thiobarbituric acid reactive substances (TBARS). (F) Representative photographs of oleogels during storage (days 1, 3, 5, and 7). Liquid corn oil subjected to the same treatment (40 °C, 24 h) served as the control (O). Different lowercase letters indicate statistically significant differences among samples on the same storage day *(p < 0.05)*.

#### 3.7.2 G′ and G″

The storage modulus (G′) reflects the energy recovered during the shear deformation cycle, indicating the elastic or solid-like behavior of the system, whereas the loss modulus (G″) corresponds to the energy consumed and is associated with its viscous or liquid-like behavior. As shown in Fig. 4B and C, the G′ of all oleogels (O5, O6, and O7) consistently exceeds the G″ across the entire frequency range, indicating that the oleogels in this study form a three-dimensional network structure, which can exhibit solid-like characteristics (Li et al., 2024). O7 maintained a high and relatively stable G′ throughout the entire frequency range, indicating the formation of a more continuous protein network with enhanced oil-holding capacity, which is consistent with the CLSM and OHC results. However, O5 exhibited a lower G′ than O7 at low frequencies (1-10 Hz) but a higher G′ at high frequencies (10-100 Hz) (Fig. 4B), which may be associated with the presence of protein aggregates that provide greater resistance to rapid oscillatory deformation, resulting in higher storage moduli (Lan et al., 2024). As shown in Fig. 4C, the slight increase in G″ observed for all oleogels with increasing frequency may be attributed to insufficient time for the protein network to rearrange during rapid oscillatory deformation. Overall, noticeable differences in G′ but relatively small differences in G″ were observed among the oleogels, suggesting that variations in the water-to-oil volume ratio primarily affected the elastic properties of the oleogels.

### 3.8 Oxidation stability

The peroxide value (POV) is a widely used indicator of primary lipid oxidation, reflecting the accumulation of lipid hydroperoxides formed during the initial stage of oxidation. The POVs of all oleogels (O5, O6, and O7) increased with the extension of storage time (days 1, 3, 5, and 7), because the presence of oxygen inevitably leads to the oxidation of lipids (Fig. 4D). On day 1, all oleogels exhibited significantly higher POV than O. This is because lipid oxidation was accelerated during oleogel preparation. Firstly, during the preparation of the emulsion-template, high-speed homogenization not only disrupts the spatial distribution of antioxidant components in the original liquid oil phase but also promotes oxygen diffusion into the oil phase. Moreover, during shearing, local temperature increases may further accelerate lipid oxidation. A similar phenomenon occurred during the preparation of whey protein isolate-carboxymethyl chitosan oleogel (Antarctic krill oil) using the emulsion-templated method (Zhao et al., 2025). However, from day 3 to day 7, all oleogels exhibited lower POV values than O, suggesting that the three-dimensional protein network enhanced oxidative stability during storage by acting as a physical barrier that restricts oxygen diffusion and lipid mobility.

Thiobarbituric acid reactive substances (TBARS) are a secondary indicator of lipid oxidation and reflect the accumulation of low-molecular-weight carbonyl compounds, particularly malondialdehyde, mainly formed during the decomposition of lipid hydroperoxides. From day 1 to day 5, all oleogels exhibited higher TBARS values than liquid corn oil, as shown in Fig. 4E. Although the protein network in the oleogel could partially restrict oxygen diffusion and thereby reduce the formation of lipid hydroperoxides, the hydroperoxides that were generated may have decomposed more readily into secondary oxidation products, leading to higher TBARS values. Moreover, interactions between proteins and lipid oxidation products within the oleogel may have further influenced the formation and accumulation of TBARS (Geng et al., 2023). O7 showed the lowest TBARS content on day 7. This may be related to the oil phase volume ratio, at which corn silk protein formed a relatively stable, continuous, and uniform network structure in O7 (as shown in CLSM images, Fig. 3A). Such a network could effectively immobilize the entrapped oil, thereby reducing lipid oxidation during storage (Rivero Meza et al., 2024). Overall, CSP-based oleogels showed relatively favorable primary oxidation behavior at some storage points, whereas consistent suppression of secondary oxidation products was not observed. These results suggest that the oleogel network may influence lipid oxidation during storage. Moreover, an appropriate oil volume fraction is a critical determinant of the oxidative stability of oleogels, as it affects protein distribution at the oil-water interface, which in turn affects the formation and integrity of the protein network.

### 3.9 Analysis of the potential mechanism for preparing plant protein-based oleogels using the emulsion-templated method

During the preparation of oleogels using the emulsion-templated method (oven-drying), plant proteins serve as both emulsifiers and the building blocks of the three-dimensional network. The performance of the final oleogels depends not only on protein-protein interactions but also on the stability of the protein-oil interface interaction.

Firstly, the construction of the emulsion template is based on the amphiphilicity of corn silk protein, which allows it to adsorb at the oil-water interface. Specifically, the hydrophobic groups are preferentially distributed towards the oil phase, while the hydrophilic groups are exposed to the water phase, forming a protein interfacial film at the oil-water interface (Fig. 5). In this study, pH 11 treatment, possibly through partial unfolding of corn silk protein, was associated with improved emulsifying properties, enabling the formation of a stable oil-in-water emulsion for subsequent oleogel preparation.

**Fig. 5:**
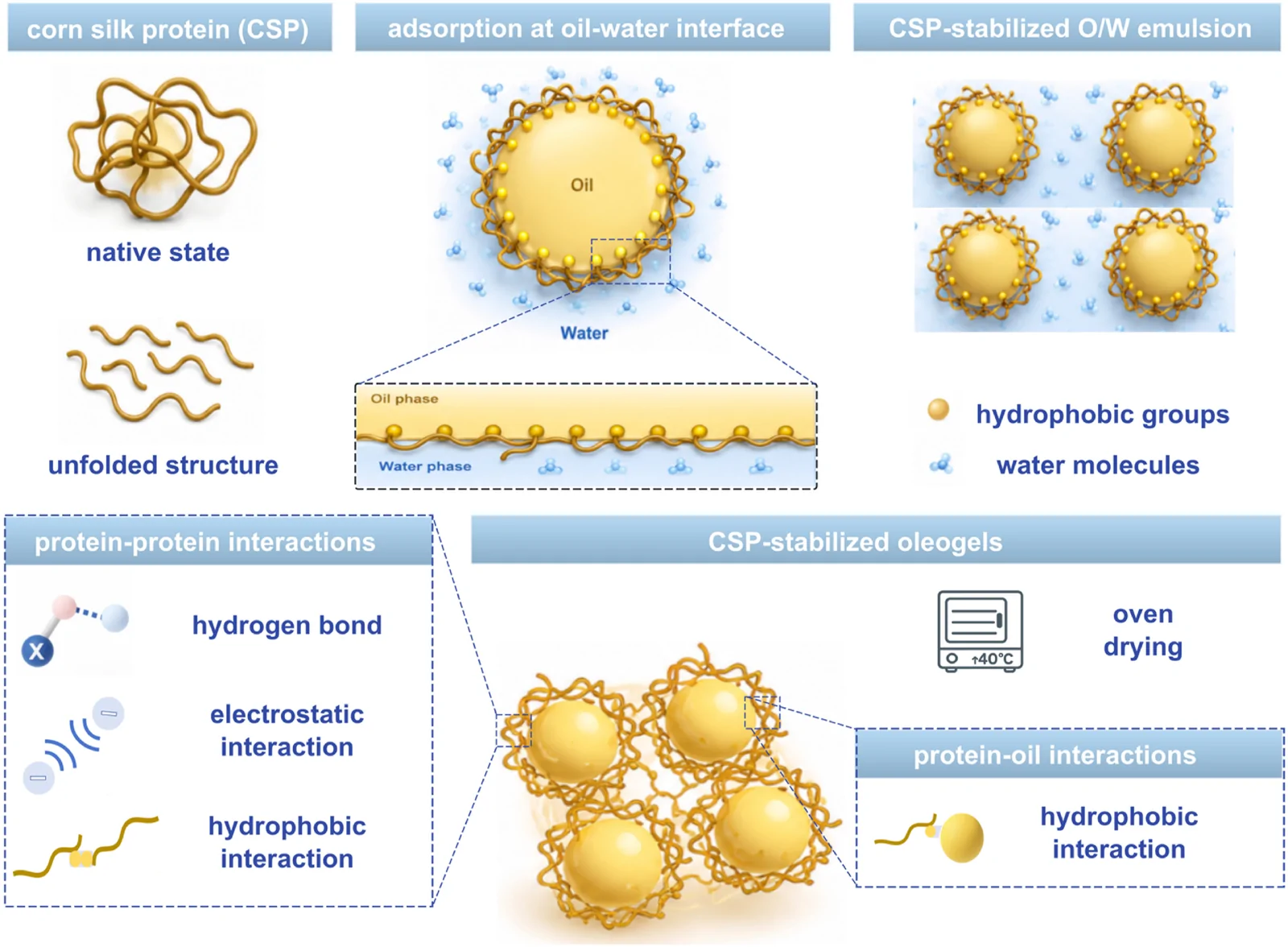
Proposed formation mechanism of corn silk protein-based oleogels fabricated via the emulsion-templated method, highlighting pH-induced protein unfolding, exposure of hydrophobic domains, and the subsequent protein-protein and protein-oil interactions responsible for oleogel network formation.

In the second stage, as water was gradually removed from the emulsion system during drying, the reduced intermolecular distance facilitated protein-protein interactions, promoting structural rearrangement and the formation of a continuous solid network. These intermolecular interactions among proteins, including hydrophobic interactions, hydrogen bonding, electrostatic interactions, and potentially disulfide exchange and/or disulfide-bond formation, regulate protein aggregation and structural rearrangement. As drying proceeds, protein films adsorbed at the oil-water interface become progressively interconnected through intermolecular interactions, resulting in the formation of a continuous three-dimensional network. Additionally, progressive water removal during drying reduces droplet separation, resulting in oil entrapment within the protein network, where liquid oil is immobilized through residual interfacial adsorption, capillary confinement, and hydrophobic interactions between proteins and oil. In summary, plant protein-based oleogels prepared by the emulsion-templated method are essentially protein-structuring processes driven by interface-induced and non-covalent interactions.

Furthermore, the volume of the oil phase is a key factor influencing the formation of oleogels and is also a variable examined in this study. The influence of the oil phase on oleogel formation mainly involves (i) affecting the geometric arrangement of the emulsion template and interface properties, (ii) impacting the oil coverage capacity of the protein interface film, and (iii) influencing the continuity and local migration of the oil phase after drying. When the oil phase volume is low (as in O5 and O6 in this study), the protein network can effectively stabilize the oil droplets; however, the relatively loose protein network provided insufficient physical entrapment for the oil phase. As the oil phase volume reaches an appropriate level (O7), proteins can form a more stable interfacial film around oil droplets and, upon drying, develop a more continuous and integrated three-dimensional network scaffold. In contrast, when the oil phase becomes excessive (O8 and O9), the available protein coverage per unit oil phase volume is no longer sufficient, leading to interfacial film thinning, increased oil droplet aggregation, and ultimately structural discontinuities in the resulting oleogels. Therefore, future studies and practical applications should explicitly define and rationally select the oil phase volume fraction when designing plant protein-based oleogels, as it plays a decisive role in governing network continuity, interfacial stability, and overall gel performance.

## 4 Conclusion

This study extracted protein from corn silk, an underutilized by-product of corn processing, and systematically investigated the effects of pH treatment on its structural and functional properties. Notably, pH 11 treatment improved functional properties associated with enhanced emulsion stabilization and allowed the successful preparation of oleogels. Corn silk protein-based oleogels were prepared using the emulsion-templated method, with a water-to-oil volume ratio of 3/7 performing best among the ratios tested in forming a stable and homogeneous protein network. However, when the oil phase exceeded 70%, oleogel formation failed, possibly due to insufficient protein available to support network formation. In contrast, lower oil fractions were associated with increased protein aggregation, reduced network uniformity, and oil droplet coalescence.

Despite these promising characteristics, such as hardness and oil-holding capacity, it should be noted that the CSP obtained by alkaline extraction and acid precipitation still contained residual non-protein components, particularly carbohydrates, which may potentially influence its emulsification, oxidation stability, and oleogel formation. Therefore, further purification of CSP to increase protein purity would be beneficial for more precisely elucidating the underlying structure-function relationship and oleogel formation mechanisms in future studies. In addition, molecular-level analyses such as circular dichroism, intrinsic fluorescence, and molecular weight distribution would help verify the pH-induced structural changes proposed in this study. Overall, this study investigates an underutilized source of plant protein and explores the structure and properties of plant protein-based oleogels, thus providing a foundation for their application in food systems, particularly for fat reduction, nutritional enhancement, and the delivery of lipid-soluble bioactive substances.

## CRediT author contributions

Chuxin Ke: Data curation; Formal analysis; Methodology; Writing - Original Draft; Investigation; Visualization.

Jiayang He: Investigation; Data curation.

Fanyu Hao: Investigation; Data curation.

Bo Pang: Conceptualization; Writing - Review & Editing; Supervision; Funding acquisition.

## Declaration of competing interest

The authors declare that they have no known competing financial interests or personal relationships that could have appeared to influence the work reported in this paper.
