# Supplementary material for: Valorization of corn silk protein: pH-driven structural modulation for emulsion-templated oleogel fabrication
Source: Curr Res Food Sci. 2026 Oct 22;13:101607. doi: 10.1016/j.crfs.2026.101607 (PMC13737722; doi:10.1016/j.crfs.2026.101607)
Supplement: Supplementary file 1 [file mmc1.docx]

**Figure S1.** Macroscopic appearances of emulsions with different water-to-oil volume ratios (5/5, 4/6, 3/7, 2/8, and 1/9), denoted as E5, E6, E7, E8, and E9, respectively.

**Table S1.** Average particle size and zeta-potential of corn silk protein-stabilized emulsions prepared at different water-to-oil volume ratios. Emulsions with water-to-oil volume ratios of 5/5, 4/6, 3/7, 2/8, and 1/9 were noted as E5, E6, E7, E8, and E9.

| **Samples** | **Average Particle Size (μm)** | **Zeta-potential (mV)** |
| --- | --- | --- |
| **E5** | 0.81±0.10^a^ | -26.87±5.13^c^ |
| **E6** | 0.93±0.05^a^ | -39.14±3.16^b^ |
| **E7** | 1.10±0.03^a^ | -49.51±2.97^a^ |
| **E8** | 4.45±0.13^b^ | -49.02±1.06^a^ |
| **E9** | 24.58±3.03^c^ | -40.15±5.96^b^ |

Data are mean ± SD of three replicates or more.

Means with different superscript letters within a column are significantly different (*p<0.05*).
